# Seven-tesla magnetic resonance imaging of the nervus terminalis, olfactory tracts, and olfactory bulbs in COVID-19 patients with anosmia and hypogeusia

**DOI:** 10.3389/fradi.2024.1322851

**Published:** 2024-10-01

**Authors:** Claudia F. E. Kirsch, Syed Ali Khurram, Daniel Lambert, Puneet Belani, Puneet S. Pawha, Akbar Alipour, Shams Rashid, Mackenzie T. Herb, Sera Saju, Yijuan Zhu, Bradley N. Delman, Hung-Mo Lin, Priti Balchandani

**Affiliations:** ^1^ Yale Department of Radiology and Biomedical Imaging, Yale School of Medicine, New Haven, CT, United States; ^2^ The School of Clinical Dentistry, University of Sheffield, Sheffield, United Kingdom; ^3^ Biomedical Engineering and Imaging Institute, Icahn School of Medicine at Mount Sinai, New York, NY, United States; ^4^ Department of Diagnostic, Molecular and Interventional Radiology, Icahn School of Medicine at Mount Sinai, New York, NY, United States; ^5^ Yale Center for Analytical Sciences, Yale School of Public Health, New Haven, CT, United States

**Keywords:** nervus terminalis (NT), olfactory tract, magnetic resonance imaging (MRI), hypothalamus, angiotensin-converting enzyme 2 (ACE-2) receptor, immune response

## Abstract

**Introduction:**

Linking olfactory epithelium to the central nervous system are cranial nerve 1, the olfactory nerve, and cranial nerve “0,” and the nervus terminalis (NT). Since there is minimal expression of angiotensin-converting enzyme-2 (ACE-2) in the olfactory nerve, it is unclear how SARS-CoV-2 causes anosmia (loss of smell) and hypogeusia (reduction of taste). In animal models, NT expresses ACE-2 receptors, suggesting a possible SARS-CoV-2 viral entry site in humans. The purpose of this study was to determine whether ultra-high-field 7 T magnetic resonance imaging (MRI) could visualize the NT, olfactory bulbs (OB), and olfactory tract (OT) in healthy controls and COVID-19 anosmia or hypogeusia and to qualitatively assess for volume loss and T2 alterations.

**Methods:**

In this study, 7 T MRI was used to evaluate the brain and olfactory regions in 45 COVID-19 patients and 29 healthy controls. Neuroimaging was qualitatively assessed by four board-certified neuroradiologists who were blinded to outcome assignments: for the presence or absence of NT; for OB, OT, and brain volume loss; and altered T2 signal, white matter T2 hyperintensities, microhemorrhages, enlarged perivascular spaces, and brainstem involvement.

**Results:**

NT was identifiable in all COVID-19 patients and controls. T2 hyperintensity in the NT, OB, and OT in COVID-19 patients with anosmia or hypogeusia was statistically significant compared to controls and COVID-19 patients without anosmia or hypogeusia.

**Discussion:**

On 7 T MRI, NT was radiographically identifiable, adjacent to OB and OT. In COVID-19 anosmia and hypogeusia, T2 hyperintensity of NT, OB, and OT was statistically significant compared to COVID-19 patients without anosmia or hypogeusia and controls. The NT may be a potential entry site for SARs-CoV-2 and may play a role in the pathophysiology of COVID-19 anosmia.

## 1 Introduction

**Figure 1:**
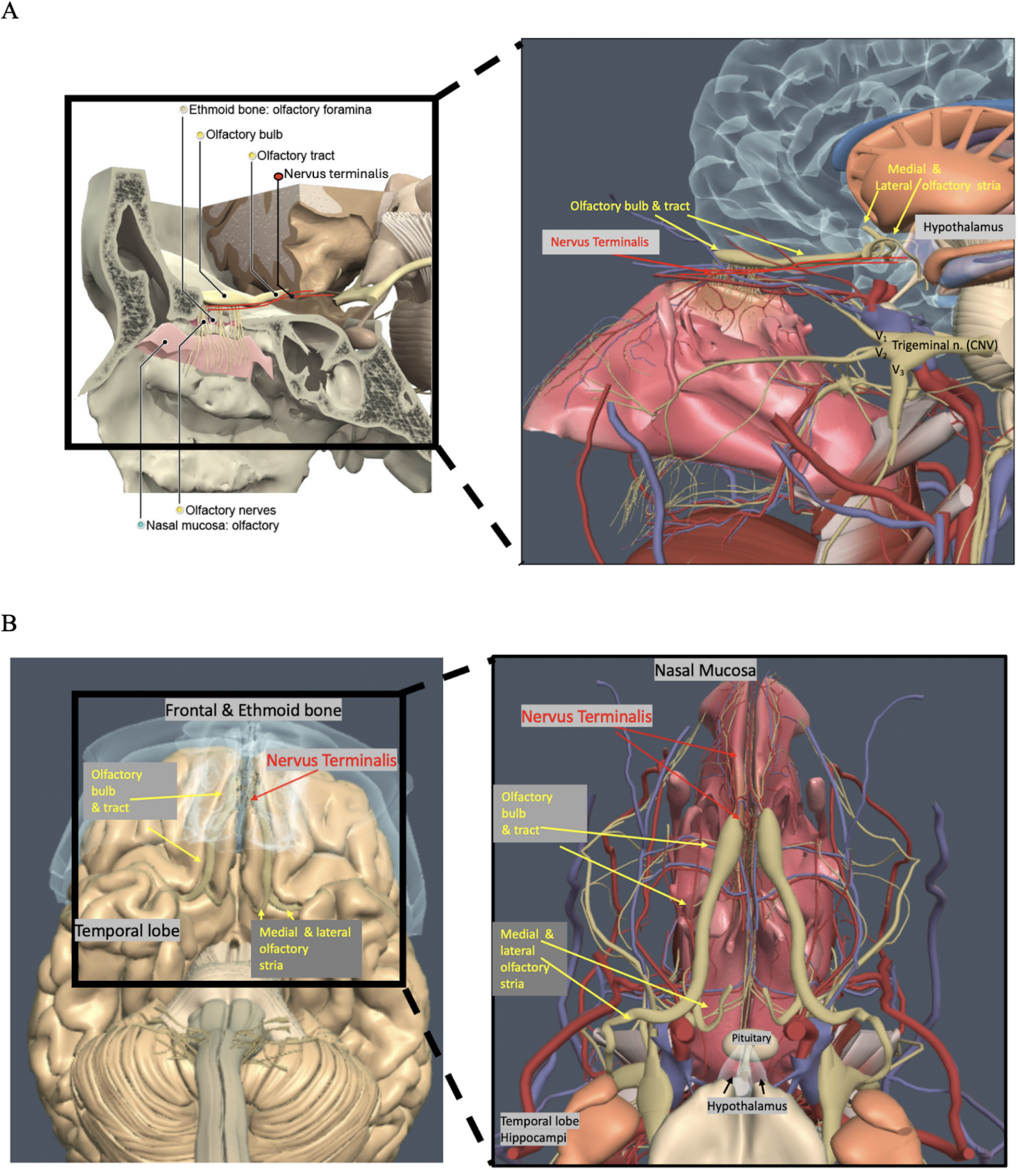
**(A,B)** Anatomy of the nasal cavity, olfactory apparatus including the olfactory bulbs (OBs), olfactory tracts (OTs), and nerves (yellow circles), nervus terminals (NT) (red oval, red lines, red arrows), nasal mucosa (tea-colored circle), and ethmoid bone and foramina (beige circle) (C. Kirsch, Primal pictures 3D anatomy). With permission © Pharma Intelligence UK (trading as Primal Pictures), 2024. www.primalpictures.com www.anatomy.tv.

The mechanism behind COVID-19-related anosmia and hypogeusia caused by SARS-CoV-2 remains unknown. SARS-CoV-2 infectivity involves the viral spike protein binding to cells exhibiting angiotensin-converting enzyme 2 (ACE-2) receptors (1–5). The olfactory epithelium is linked to the central nervous system (CNS) via two cranial nerves: cranial nerve “0,” the nervus terminalis (NT), and cranial nerve one, the olfactory nerve, which comprises olfactory filia connecting to olfactory bulbs (OBs) and olfactory tracts (OTs); collectively with the olfactory epithelium, these structures are referred to as the olfactory apparatus (OA). Currently, no consensus exists on whether SARS-CoV-2 directly infects olfactory neural tissue (5–8). Although ACE-2 expression is noted in supporting sustentacular cells and vascular pericytes, there is no significant ACE-2 expression in the OB or OT (5–11). In comparison, the NT demonstrates ACE-2 expression; these neural fibers are composed of unmyelinated sensory and autonomic nerves, with the majority of cell bodies located along the ventromedial OB (10, 12–16). NT fibers innervate the olfactory epithelium Bowman's glands and vasculature with neuronal projections to the hypothalamus (11–16) (Figure 1).

How the SARS-CoV-2 affects the OA is currently not elucidated, especially as the OB and OT do not have ACE-2 receptors (5–7). Adjacent to the OB and OT is the NT; these unmyelinated neurons have been described in histopathologic specimens since 1914 (16–22), yet many clinicians are unaware of NT's significance due to a lack of description in the contemporary imaging literature (17). The NT in animal models demonstrates ACE-2 receptors and is a proposed site of SARS-CoV-2 viral entry (23, 24). These thin, delicate unmyelinated NT fibers are formed by intermixed terminal nerve fibers at the superior anterior nasal cavity and vomeronasal (VN) fibers, which innervate Bowman’s gland cells and *vessels* (19, 23, 24). In the literature, NT fibers are referred to by various names, including CN 0 or 13, the nerve of Pinkus, *tractus olfacto-commissuralis*, new or terminal nerve, and *nerve nulla* (12, 15–17). The NT was officially designated in 1998 as CN 0 in the *Terminología Anatómica* by the Federative Committee on Anatomical Terminology of the International Federation of Associations of Anatomists (25). NT fibers extend from the cribriform plate to the olfactory trigone, septal nuclei, supraoptic hypothalamic nucleus, and hippocampus. Studies have demonstrated that luteinizing hormone-releasing hormone (LHRH) cells migrate via the NT from neural crest olfactory placodes to the hypothalamus (26–28). Innervation to the hypothalamus by the NT presents a potential route of viral transmission to the hypothalamus, with recent studies demonstrating hypothalamic–pituitary axis alterations in COVID-19 (28, 29). The absence of migratory LHRH cells is thought to be the etiology of Kallmann's syndrome, a form of hypogonadism associated with absent or small olfactory bulbs and anosmia (30).

The NT is highly conserved in all vertebrates, including whales and dolphins, who lack olfactory systems (17, 20, 22, 31, 32). The NT is involved in immune responses and produces nitric oxide (NO), leading to vasodilation (32–34). At higher concentrations, NO can lead to apoptotic cell death through the indirect activation of caspases (35–38). SARS-CoV-2 involving the NT may possibly affect nitric oxide release, affecting the vasculature and possibly causing programmed neuronal cell death (34–38).

The delicate NT fibers are often overlooked in imaging research because of the small size in the anterior skull base, alongside the OB and OT (17, 39). Although NT fibers have been well illustrated in human pathologic literature since 1914 (18, 21, 22), their exclusion from medical curricula and easy disruption during autopsies contribute to a lack of current clinical awareness (15–17). Importantly, animal models have revealed that the NT fibers express ACE-2 receptors (23, 24). ACE-2 receptors along the NT may allow for SARS-CoV-2 viral entry, possibly affecting the vascular supply to the OA, including the OB and OT, and allow for viral entry to the hypothalamus. Although 1.5- and 3-T MRI scans have demonstrated OB and OT alterations in COVID-19 patients with anosmia, including volume loss and T2 hyperintensity (39–45), the mechanism behind these changes remains unknown due to the lack of ACE-2 receptors in the OB and OT (5). MRI imaging of the OB, OT, and NT at 3 T and lower field strengths is limited by the small size of these structures and their anterior skull base location, where air tissue interfaces can create artifacts (39, 40, 46). The purpose of this study was to determine whether 7-T MRI could visualize the NT, OB, and OT in healthy controls and COVID-19 patients and to detect volume alterations and/or increased T2 signal intensity in the NT, OB, and OT in COVID-19 patients with anosmia or hypogeusia.

## 2 Materials and methods

### 2.1 Study design

In this case–control, cross-sectional imaging study, 7-T MRI was used to evaluate the brain and olfactory region in 45 COVID-19 patients and 29 healthy controls. The neuroimaging was qualitatively assessed, initially individually and then via consensus, by four board-certified neuroradiologists who were blinded to group and outcome assignments; they evaluated the presence or absence of the NT in both healthy controls and COVID-19 patients. In addition, each neuroradiologist qualitatively assessed the MRI images for volume loss and altered T2 signals in the NT, OB, and OT, as well as for brain parenchymal volume loss, white matter T2 hyperintensities, microhemorrhages, enlarged perivascular spaces, and brainstem involvement. Institutional Review Board approval was obtained prior to recruitment, and written and informed consent was obtained from all subjects before MRI scanning.

### 2.2 Participants

Patient demographics of healthy controls and COVID-19 patients are presented in Table 1. From March 2021 to July 2023, 74 subjects were scanned, including 45 COVID-19 patients, who were recruited via referrals by treating physicians and neurologists. A total of 29 age- and gender-matched controls were recruited by physician referrals and the researchmatch.org website. The period between initial COVID-19 diagnosis and obtaining the 7-T MRI scan was available in a subset of 37 COVID-19 patients, with an average of 21 ± 9 months, ranging from a minimum of 2 months to a maximum of 36 months. Inclusion criteria for COVID-19 patients included age 18 years or older, ability to speak and understand English, recovery from COVID-19 with moderate respiratory and/or neurological symptoms, and recovery from COVID-19 with severe respiratory symptoms requiring ventilation. COVID-19 patients were recruited based on the following criteria: presence of one or more COVID-related neurological symptoms such as brain fog, impaired memory, headache, altered taste/smell, fatigue, depression, anxiety, hallucinations, speech disorders, visual abnormalities, and seizures/convulsions during or after acute COVID-19 infection. Patients with Parkinson's disorder, history of agitation, apathy, depression, anxiety, hallucinations, and personality disorders were excluded. Age- and gender-matched healthy control subjects were recruited and enrolled if they met one of the following criteria: (A) no prior COVID-19 infection or (B) asymptomatic or mildly symptomatic COVID-19, not requiring medication or physician evaluation more than 12 months prior to the study, no neurological symptoms, and no prior hospitalizations for COVID-19. All subjects undergoing a 7-T MRI brain scan were screened for MRI eligibility, and any subjects with ferromagnetic or non-MRI-compatible implants or devices, claustrophobia, and pregnancy were excluded. COVID-19 disease severity was assessed using the National Institute of Health (NIH) COVID Treatment Guidelines Panel (https://www.covid19treatmentguidelines.nih.gov/) (47).

**Table 1:** Comparison of demographics and 7-T MRI findings in the NT, OBs, OTs, and brain white matter between COVID-19 patients and healthy controls.

Variable	COVID-19 patients	Healthy controls	*p*-value^a^
*N*	45	29	
Age (years)	47 ± 13	42 ± 13	
Gender, male (%)	16 (35.6%)	15 (51.7%)	
Gender, female (%)	29 (64.4%)	14 (48.3)	
No. of COVID-19 patients receiving hospital treatment	10 (22.2%)		
No. of COVID-19 patients with anosmia during initial COVID-19 infection	26/45 = 57.8%		
No. of COVID-19 patients with hypogeusia during initial COVID-19 infection	26/45 = 57.8%		
No. of COVID-19 patients with anosmia and hypogeusia during initial COVID-19 infection	23/45 = 51.1%		
No. of COVID-19 patients with anosmia, with no hypogeusia during initial COVID-19 infection	3/45 = 6.7%		
No. of COVID-19 patients with hypogeusia, with no anosmia during initial COVID-19 infection	3/45 = 6.7%		
No. of COVID-19 patients with anosmia during and after recovery from COVID-19 infection	13/45 = 28.9%		
No. of COVID-19 patients with anosmia after recovery from COVID-19 infection	14/45 = 31.1%		
No. of COVID-19 patients with anosmia after recovery, no anosmia during COVID-19 infection	1/45 = 2.2%		
No. of COVID-19 patients with hypogeusia after recovery from COVID-19 infection	12/45 = 26.7%		
No. of COVID-19 patients with hypogeusia after recovery, no hypogeusia during COVID-19 infection	2/45 = 4.4%		
No. of COVID-19 patients with anosmia or hypogeusia during or after COVID-19 infection	29/45 = 64.4%		
No. of COVID-19 patients with no anosmia or hypogeusia during or after COVID-19 infection	16/45 = 35.5%		
No. of COVID-19 patients with T2 hyperintensity in the NT, OB, and OT	25/45 = 51.0%		
No. of COVID-19 patients with anosmia with T2 hyperintensity in the NT, OB, and OT	22/26 = 84.6%		<0.0001^b^
No. of COVID-19 patients without anosmia with T2 hyperintensity in the NT, OB, and OT	3/19 = 15.8%		
No. of COVID-19 patients with anosmia or hypogeusia with T2 hyperintensity in the NT, OB, and OT	23/29 = 79.3%		<0.0001^c^
No. of COVID-19 patients without anosmia or hypogeusia with T2 hyperintensity in the NT, OB, and OT	2/16 = 12.5%		
0–4 scale of T2 hyperintensity in white matter on 7-T MRI			
0 = absent	21/45 = 53%	22/29 = 75.9%	0.040^a^
1 = minimal punctate foci periventricular and deep white matter	20/45 = 44%	6/29 = 20.7%	0.047^a^
2 = periventricular white matter halo lesions and confluent lesions in deep white matter	4/45 = 8.9%	1/29 = 3.4%	0.642^a^

^a^Fisher's exact test.

^b^ T2 hyperintensity for COVID-19 patients with anosmia (84.6%) vs. controls (0%) and COVID-19 patients without anosmia (15.8%) are statistically significant (Fisher's exact test, *p* < 0.0001 for both).

^c^ T2 hyperintensity for COVID-19 patients with anosmia or hypogeusia (79.3%) vs. controls (0%) and COVID-19 patients without anosmia or hypogeusia (12.5%) are statistically significant (Fisher's exact test, *p* < 0.0001 for both).

### 2.3 7-T MRI protocol

Images of the brain and olfactory region were acquired at the Icahn School of Medicine, BioMedical Engineering and Imaging Institute using a Siemens 7-T MAGNETOM system scanner (Siemens Healthineers GmbH, Erlangen, Germany) equipped with 70 mT/m, 200 T/m/s gradients and a single-channel transmit, 32-channel receive head coil (Nova Medical, Wilmington, MA, USA). The imaging protocol included localizer, static field (B_0_) shimming, and radiofrequency field (B_1_) mapping by saturation-prepared turbo fast-low-angle-shot (FLASH), followed by high-resolution anatomical, susceptibility, relaxometry, and diffusion sequences of the brain, olfactory tract, and olfactory bulbs. T1 and T2 relaxometry were performed using magnetization-prepared 2-rapid gradient-echo (MP2RAGE) and turbo spin-echo (TSE) at multiple echo times (TE), respectively, followed by diffusion MRI using spin-echo EPI and susceptibility-weighted imaging (SWI) with ASPIRE, processed via the CLEAR-SWI package (Table 2) (48, 49).

**Table 2:** 7-T MRI sequences for the brain and olfactory apparatus.

Sequence	TR (ms)	TE (ms)	Inversion time (ms)	Flip angle (°)	Matrix size (X × Y × Z)	Voxel size (mm^3^)	*b*-value (s/mm^2^)	Diffusion directions
3D isotropic MP2RAGE	9,000	5.1	IT1: 1,050 IT2: 3,000	FA1: 5 FA2: 4	320 × 240 × 240	0.7 × 0.7 × 0.7	—	—
T2-TSE	6,000	69	—	150	512 × 512 × 512	0.4 × 0.4 × 0.4	—	—
SWI	23	14	—	12	1,024 × 864 × 80	0.2 × 0.2 × 1.5	—	—
DWI	7,200	68	—	90	200 × 200 × 66	1 × 1 × 1	1,500	64

TR, time repetition; TE, time to echo; SWI, susceptibility-weighted imaging; DWI, diffusion weighted imaging.

### 2.4 7-T MRI data analysis

MRI scans of the brain parenchyma, NT, OB, and OT of all healthy controls and COVID-19 patients were reviewed by four board-certified neuroradiologists, each holding current neuroradiology certificates of added qualification (CAQ) (PBe, PP, BD, CK) with 10–22 years of experience. Each neuroradiologist reviewed scans individually and then via a consensus review, blinded to clinical status, with careful consensus re-review assessing for the presence or absence of the NT in all cases; the presence or absence of T2 hyperintensity in the NT, OB, or OT; volume loss in the OB, OT, or brain; T2 white matter hyperintensities; hemorrhages, brainstem involvement; and enlarged perivascular spaces. Brain white matter T2 hyperintensity was graded on a 0–4 scale; 0 = absent 1 = minimal punctate foci periventricular and deep white matter; 2 = periventricular white matter halo lesions and confluent lesions in deep white matter; 3 = irregular periventricular white matter signal extending to deep white matter, confluent deep white matter lesions; and 4 = extensive confluent periventricular and deep white matter abnormalities. After individual and consensus reviews were completed, imaging results were tabulated and correlated with COVID-19 status, patient age, biological sex, presence or absence of anosmia, phantosmia, and hypogeusia during initial COVID-19 infection or persisting after recovery, and whether patients were hospitalized or treated at home.

### 2.5 Statistics

SPSS (version 29.0) software was used for all statistical calculations. A sample *t*-test was used to compare age differences in COVID-19 patients and controls. A chi-square was utilized to compare gender differences in COVID-19 patients and controls, with no significant differences observed per sample size. Fisher's exact test was conducted to test for significant differences in T2 hyperintensity in the NT, OB, and OT in COVID-19 patients with anosmia or hypogeusia during initial COVID-19 infection or persisting after recovery from COVID-19 and was compared to healthy controls and COVID-19 patients without anosmia or hypogeusia. Statistical significance was set at *p*-values ≤0.05 to test whether the null hypothesis should be rejected. A careful consensus re-review was performed to check for inter-rater reliability for T2 hyperintensity in the NT, OB, and OT with all four board-certified neuroradiologists blinded to clinical status, with achieved consensus agreement on determining the presence of T2 hyperintensity in the NT, OB, and OT, as well as volume loss, T2 white matter changes, microhemorrhages, and enlarged perivascular spaces. Data from the consensus review were correlated with clinical status and used for statistical analyses.

## 3 Results

A total of *n* = 45 COVID-19 patients were scanned using 7-T MRI. Among them, *n* = 26 COVID-19 patients reported anosmia during initial COVID-19 infection, *n* = 23 reported both anosmia and hypogeusia, and *n* = 3 reported anosmia without hypogeusia. In addition, *n* = 14 patients experienced persistent anosmia after recovery from COVID-19 infection, of whom *n* = 13 had prior anosmia during initial COVID-19 infection, while only 1 patient reported developing anosmia after recovery from COVID-19, having had no anosmia at the time of initial COVID-19 infection. A total of *n* = 26 patients reported hypogeusia at initial COVID-19 infection, with *n* = 3 reporting hypogeusia without anosmia at the time of initial COVID-19 infection and *n* = 12 reporting hypogeusia after recovery from COVID-19, of whom *n* = 10 had experienced hypogeusia initially and *n* = 2 developed hypogeusia without hypogeusia at time of initial COVID-19 infection (Table 1).

**Figure 2:**
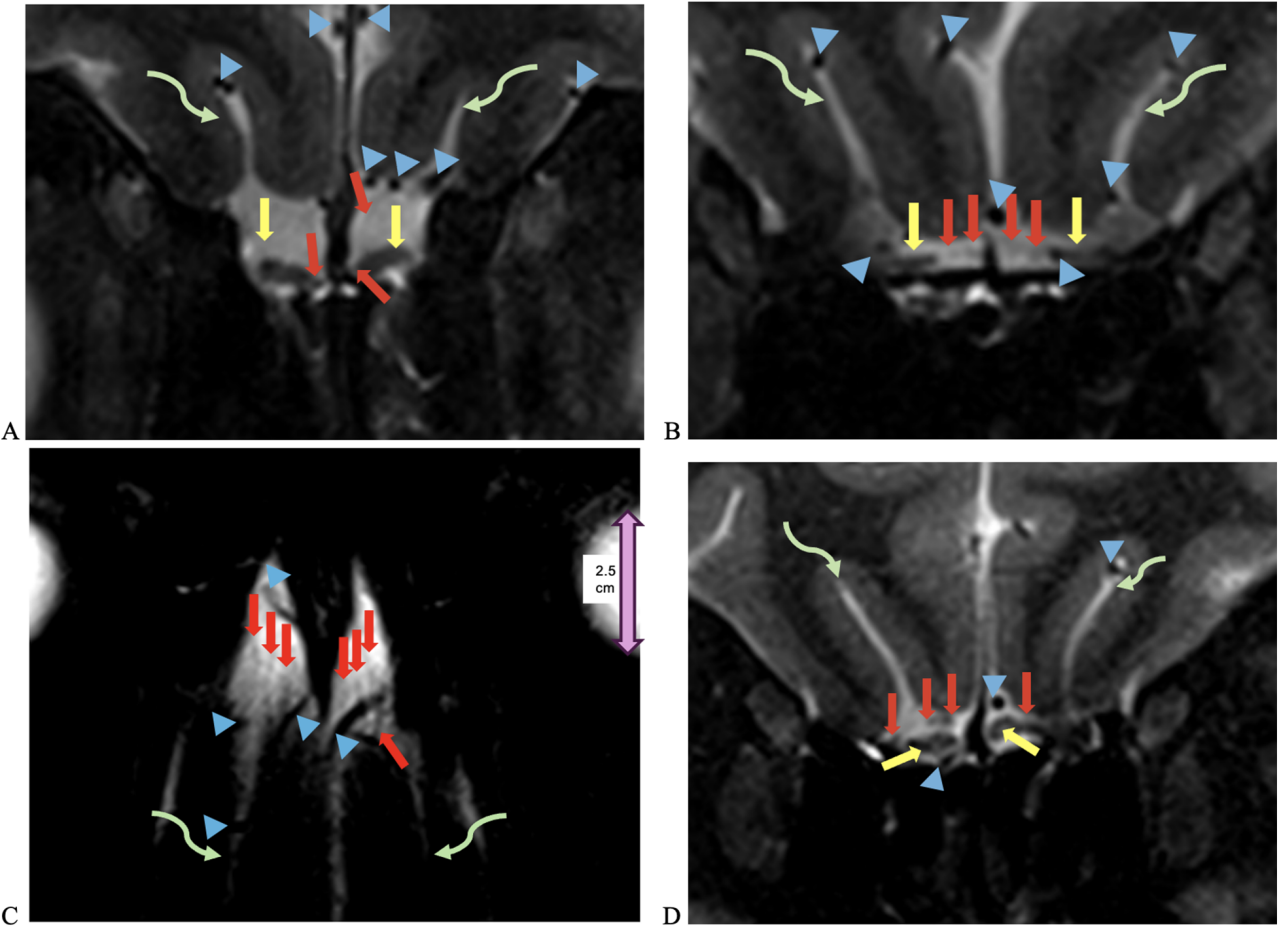
UHF 7-T MRI T2-weighted **(A,B)** coronal and **(C)** axial scans of a 38-year-old female healthy control. In **(C)**, the orbital globe, pink dual arrow, measures 2.5 cm anterior–posterior for scale. **(D)** UHF 7-T MR T2-weighted coronal images of a 33- year-old female healthy control. Yellow arrows point to olfactory bulbs in **(A,D)** and olfactory tracts, red arrows point to the NT, blue arrowheads point to vessels with flow voids, and curved green arrows point to the olfactory sulcus. The depth of the olfactory sulcus in the coronal plane measures between 6.5 and 7.5 mm.

**Figure 3:**
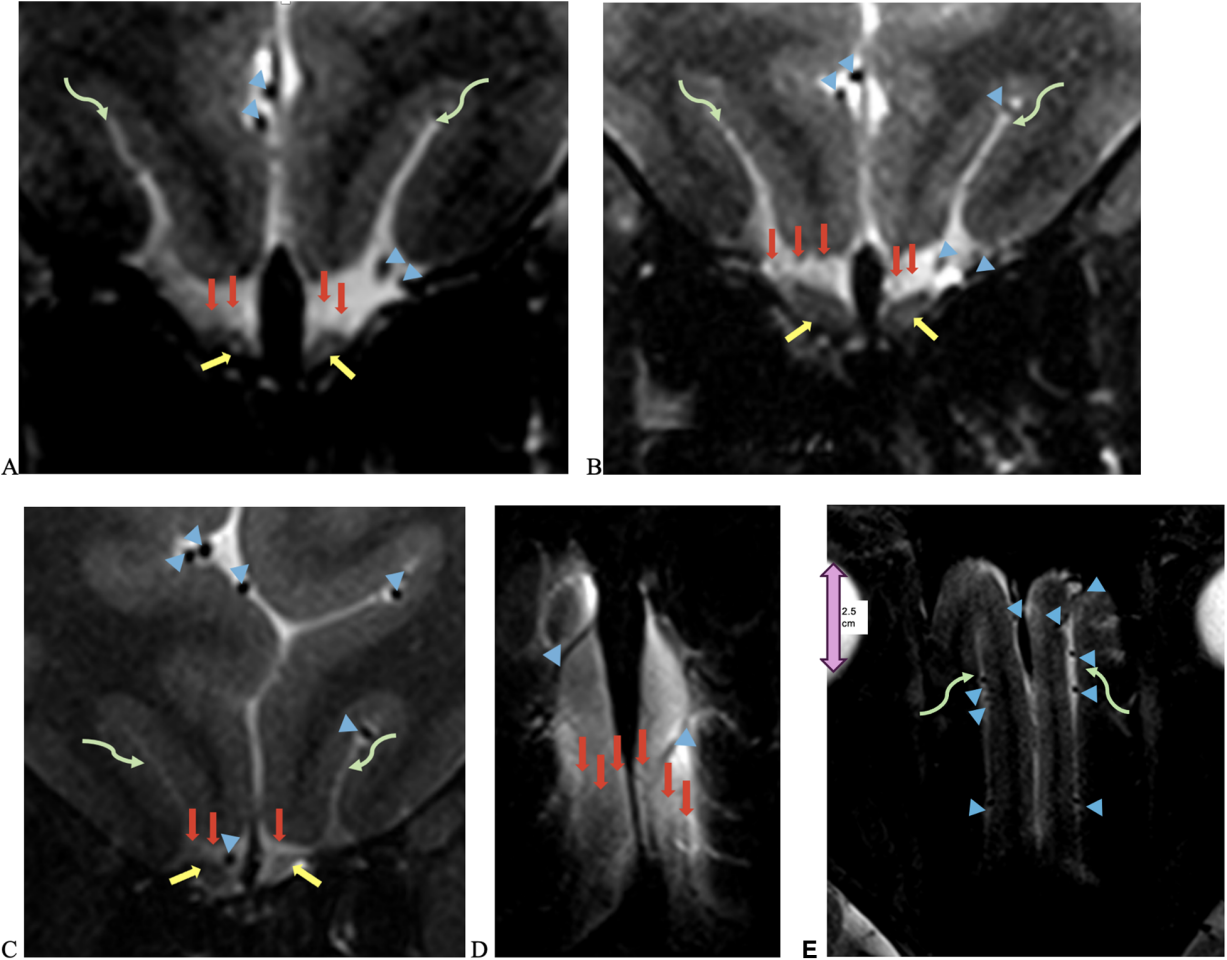
UHF 7-T MRI T2-weighted **(A–C)** coronal and **(D,E)** axial scans of a 43-year-old female with anosmia and hypogeusia during and persisting after recovery from COVID-19. Yellow arrows point to olfactory bulbs, red arrows point to the NT, blue arrowheads point to vessels with flow voids, and curved green arrows point to the olfactory sulcus. There is an increased T2 signal in the bilateral olfactory bulbs, with the left greater than right olfactory tracts and NT, as well as left volume loss greater than right volume loss in the left olfactory bulb and tract, with an asymmetric widening of the left olfactory sulcus.

The NT was identifiable on all 7-T MRI scans, visualized on coronal and axial T2-weighted sequences adjacent to the OB and OT as curvilinear structures of a non-fluid signal bathed in the T2 hyperintensity of surrounding cerebrospinal fluid (CSF), in all healthy controls and COVID-19 patients with and without anosmia and hypogeusia (Figures 2, 3). On axial and coronal T2-weighted images, the NT appeared as thin, linear, non-vascular structures in the expected location reported in the histopathological literature adjacent to the OB and OT (Figures 2, 3). On 7-T MRI T2-weighted sequences, in all healthy controls and 18 of 19 COVID-19 patients without anosmia and hypogeusia, the OA demonstrated predominately qualitative decreased T2 signal without T2 hyperintensity. One unexpected finding was T2 hyperintensity in the NT, OB, and NT in one COVID-19 patient without anosmia or hypogeusia. Qualitative T2 hyperintensity was noted in the NT, OB, and OT on T2-weighted MRI scans in *n* = 22 of 26 patients with COVID-19-related anosmia (84.6%); when compared to controls at 0% and COVID-19 patients without anosmia at 15.8%, this result was statistically significant (Fisher's exact test with *p* < 0.0001 for both, Table 1). Qualitative T2 hyperintensity in the NT, OB, and OT was noted in 79.3% of COVID-19 patients with anosmia or hypogeusia, and when compared to controls at 0% and COVID-19 patients without anosmia or hypogeusia at 12.5%, this result was also statistically significant (Fisher's exact test *p* < 0.0001 for both, Table 1).

OB and OT volume loss was qualitatively assessed in COVID-19 patients with anosmia and hypogeusia during initial infection and after recovery from COVID-19; *n* = 8 (17.8%) exhibited OB and OT volume loss on independent and consensus review compared to controls; the volume loss also demonstrated ancillary findings, including enlarged gyrus rectus sulci (Figure 3). In the healthy control group, *n* = 2 (6.9%) demonstrated subjective OB and OT volume loss (Table 1). Fisher's exact test revealed no significant differences in OB and OT volume loss between COVID-19 patients with anosmia and hypogeusia vs. healthy controls or COVID-19 patients without anosmia or hypogeusia.

Among COVID-19 patients, 26.3% demonstrated qualitative brain volume loss that was either mild or greater than expected for their age, with 100% agreement on consensus review. Brain volume loss was predominantly noted in patients greater than 50 years of age in COVID-19 and healthy controls. In the COVID-19 cohort, minimal grade 1 white matter changes were noted in *n* = 20 (44%), while grade 2 periventricular white matter halo and confluent deep white matter lesions were noted in *n* = 4 (8.9%). In controls, grade 1 white matter hyperintensities were noted in *n* = 6 (20.7%) and grade 2 in *n* = 1 (3.4%). In both COVID-19 patients and controls, white matter changes correlated with increasing age (Supplementary Material Graph 1). The 7-T MRI scans of the COVID-19 cohort demonstrated 5.3% with chronic microhemorrhages, likely due to microvascular disease, and 31.6% with enlarged perivascular spaces, which were not appreciated in age-matched controls, a finding previously reported in long COVID neurologic sequelae (50). There were no acute infarcts, acute hemorrhages, or deep brainstem pathologic findings on any of the reviewed images.

## 4 Discussion

**Figure 4:**
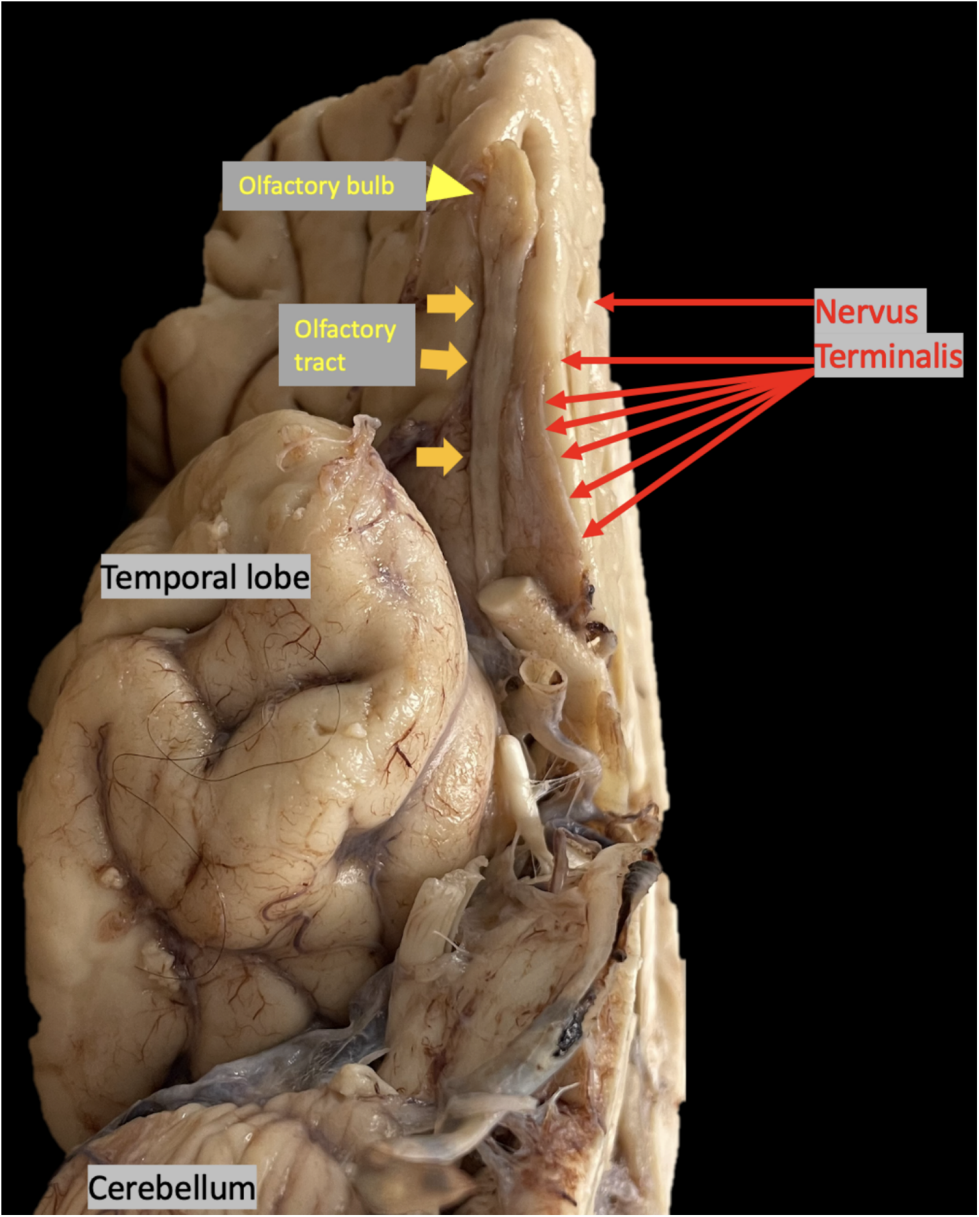
Autopsy brain specimen, yellow arrowhead points to olfactory bulb (OB), yellow arrows point to olfactory tract (OT), red arrows point to nervus terminalis (NT) the thin unmyelinated fibers adjacent to the OB and OT.

In this study, 7-T MRI T2-weighted images demonstrated thin linear structures with decreased T2 signals in the expected region of the NT, as reported in the histopathologic literature, in all healthy controls and COVID-19 patients, located adjacent to the OB and OT, distinct from vascular flow voids, as demonstrated in Figures 2–4 (18–22). This study using 7-T MRI is one of the first to radiographically demonstrate the NT in healthy controls and COVID-19 patients. The ability to identify the NT on MRI may assist researchers in future studies. Qualitative analysis of COVID-19 patients with anosmia and hypogeusia demonstrated T2 hyperintensity along the NT, OB, and OT, which was not appreciated in the healthy controls and in the majority of COVID-19 patients without anosmia. The increased T2 hyperintensity of the NT, OB, and OT in COVID-19 patients with anosmia was statistically significant with *p*-values <0.0001 in Fisher's exact test when compared to healthy controls and COVID-19 patients without anosmia (Table 1, Figures 2, 3). T2 hyperintensity in the NT, OB, and OT for COVID-19 patients with anosmia or hypogeusia (79.3%) vs. controls and COVID-19 patients without anosmia or hypogeusia was statistically significant with *p*-values <0.0001 (Fisher's exact test for both). MRI T2 hyperintensity in COVID-19 patients with anosmia along the OB has been reported in 1.5- and 3-T MRI, suggesting increased fluid signal or edema that may occur from an inflammatory or ischemic response with vasogenic or cytotoxic edema (39–44, 51–53).

In this study, T2 hyperintensity was noted in the NT, OB, and OT in COVID-19 patients with anosmia and hypogeusia; however, it has previously been reported that the OB and OT do not express ACE-2 required for SARS-CoV-2 viral entry (5). The NT, however, does express ACE-2 and is a unique mixture of sensory and autonomic nerves, with cell bodies located along the ventromedial OB, innervating the olfactory epithelium, Bowman's glands, and vasculature (23, 24). The NT fibers innervate nasal epithelial support cells and Bowman's glands, which express ACE-2; In addition, the NT fibers contain cathepsin L and B enzymes, with similar characteristics to the proteolytic activity of transmembrane protease, serine 2 (TMPRSS2), a host cell factor that aids SARS-CoV-2 cell entry (24). The presence of ACE-2 receptors along the NT is suggestive of a potential neurologic entry site for SARS-CoV-2 (10, 23, 24). Because the NT projects to the hypothalamic area, there is potential for viral intracranial spread along the NT to the hypothalamic region (7, 10, 23, 24). Since the NT expresses nitric oxide synthase in some species and may release nitric oxide and because nitric oxide is involved in inflammation and immune responses, it is possible that NT virus-induced inflammation could affect the vascular supply to the adjacent OB and OT (33–36). At the current time, research has not shown evidence of SARS-CoV-2 causing neuronal apoptosis in the OB, although microvascular pathology and downregulation of olfactory receptors and signaling components have been noted (37, 38). Additional mechanisms that may contribute to anosmia in COVID-19 include the elimination of support cells and the retraction of cilia on olfactory receptor neurons in the olfactory epithelium (7). Continued 7-T MRI and histopathologic research may better quantify these findings in COVID-19 patients with anosmia or hypogeusia.

Previous 1.5- and 3-T MRI scans of COVID-19 patients with anosmia have documented OB volume loss, increased T2 signal, and microhemorrhages (39–45). A key unanswered question is how the SARS-CoV-2 virus leads to these MRI findings of OB and OT volume loss and T2 hyperintensity. In non-human primate rhesus monkeys, it is reported that SARS-CoV-2 invades the CNS via the olfactory bulb and then spreads rapidly to the hippocampus, thalamus, and medulla oblongata (54). However, in humans, there are limited ACE-2 receptors in the OB, and although SARS-CoV-2 RNA has been detected in the CNS of patients who died from lethal COVID-19 infection, no definitive ultrastructural evidence of SARS-CoV-2 in the human CNS has been reported in the literature (5, 8). Our study utilizing 7-T MRI provided improved signal-to-noise ratios and enhanced conspicuity of the NT, OB, and OT (24–28). In this study, 7-T MRI demonstrated qualitative T2 hyperintensity along the NT, OB, and OT in COVID-19 patients with anosmia and in COVID-19 patients with anosmia or hypogeusia, which was statistically significant compared to healthy controls and COVID-19 patients without anosmia or hypogeusia.

Although the 29 healthy controls were screened and did not report anosmia or hypogeusia, 2 control MRI scans demonstrated subjective OB and OT volume loss (6.9%) on both independent and consensus reviews. OB and OT volume loss may reflect anatomic variation or prior OA pathology, which represents an additional limitation of this study. In the healthy control group, previous olfactory pathology, anosmia, or hyposmia cannot always be excluded with certainty, as noted in the literature; even prior to the COVID-19 epidemic, up to 30% of healthy individuals may have been anosmic or hyposmic (55).

Study limitations include limited sample size and potential selection bias from screening for 7-T MRI, with inadvertent exclusion of severe COVID-19 cases with intubation or marked neurologic impairment. In this study, the majority of COVID-19 patients (78%) were not hospitalized, reflected in MRI findings of no infarcts, no new hemorrhages, and no brainstem involvement. An additional limitation of this study is that OB and OT volume loss was determined qualitatively, without quantitative volumetric measurements. Because quantitative measurements were not performed, slight symmetric OB or OT volume loss may have been under-reported. Additional research with quantitative measurements may have appreciated slight OB or OT volume loss that was not readily noticed through qualitative assessments. The outcomes of this study may warrant future research with quantitative assessments of the NT, OB, and OT.

In this study, 7-T MRI delineated linear structures adjacent to the OB and OT, corresponding to the NT, as illustrated in histopathologic studies. A qualitative assessment of T2 hyperintensity in the NT, OB, and OT was statistically significant in COVID-19 patients with anosmia and COVID-19 patients with anosmia or hypogeusia compared to healthy controls and COVID-19 patients without anosmia or hypogeusia. Further research with quantitative measurements of OB and OT volumes, T2 hyperintensity, imaging larger numbers of patients, and histopathologic correlation with brain bank specimens may better delineate how the NT, OB, and OT are affected by SARS-CoV-2 and the mechanisms involved in COVID-19-related anosmia or hypogeusia.

## Data Availability

The raw data supporting the conclusions of this article will be made available by the authors without undue reservation.
