# Supplementary figures and images for: Seven-tesla magnetic resonance imaging of the nervus terminalis, olfactory tracts, and olfactory bulbs in COVID-19 patients with anosmia and hypogeusia
Source: Front Radiol. 2024 Oct 1;4:1322851. doi: 10.3389/fradi.2024.1322851 (PMC11473298; doi:10.3389/fradi.2024.1322851)

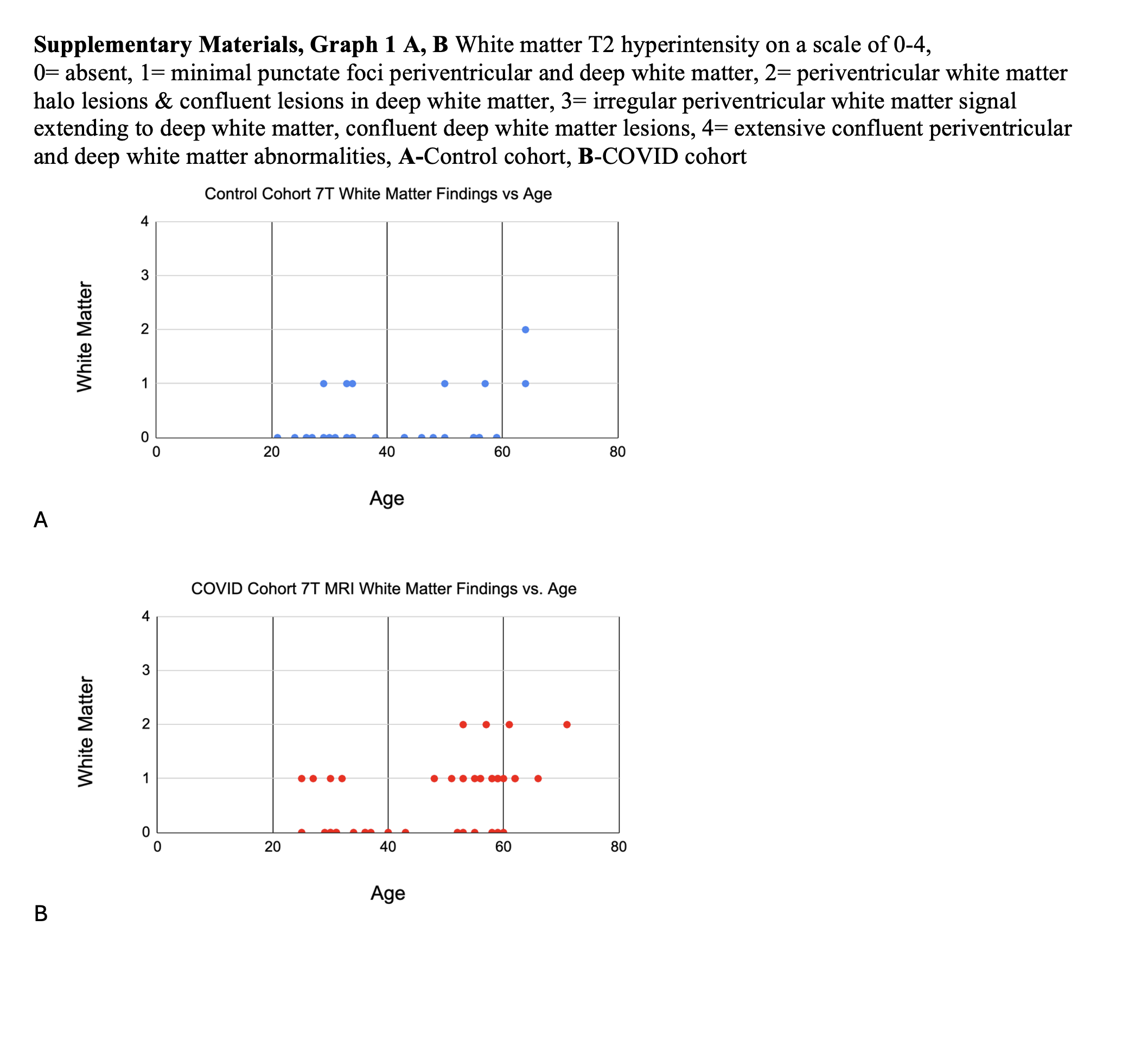

Supplement: Supplementary file 1 [file Image1.tiff]
